# Agile science: creating useful products for behavior change in the real world

**DOI:** 10.1007/s13142-016-0395-7

**Published:** 2016-02-26

**Authors:** Eric B. Hekler, Predrag Klasnja, William T. Riley, Matthew P. Buman, Jennifer Huberty, Daniel E. Rivera, Cesar A. Martin

**Affiliations:** School of Nutrition and Health Promotion, Arizona State University, 500 N. 3rd Street, Phoenix, AZ 85003 USA; University of Michigan, Ann Arbor, MI 48109 USA; National Institutes of Health, Bethesda, MD 20892 USA

**Keywords:** Behavior change, Implementation science, Research methods

## Abstract

Evidence-based practice is important for behavioral interventions but there is debate on how best to support real-world behavior change. The purpose of this paper is to define products and a preliminary process for efficiently and adaptively creating and curating a knowledge base for behavior change for real-world implementation. We look to evidence-based practice suggestions and draw parallels to software development. We argue to target three products: (1) the smallest, meaningful, self-contained, and repurposable behavior change modules of an intervention; (2) “computational models” that define the interaction between modules, individuals, and context; and (3) “personalization” algorithms, which are decision rules for intervention adaptation. The “agile science” process includes a generation phase whereby contender operational definitions and constructs of the three products are created and assessed for feasibility and an evaluation phase, whereby effect size estimates/casual inferences are created. The process emphasizes early-and-often sharing. If correct, agile science could enable a more robust knowledge base for behavior change.

## INTRODUCTION

A central task of behavior change science is to generate evidence to support the development of evidence-based practices [1]. Evidence-based practices not only require careful examination of the efficacy of an intervention but also the ability of the evidence-based practice to be translated and disseminated for real-world use [2]. There is active discussion on how to improve the research process for achieving real-world evidence-based practice such as a more rapid, relevant, and responsive research enterprise; building on the logic of “disruptive innovations;” placing greater emphasis on the value of the components of a multicomponent “complex” intervention; and placing greater emphasis on the need for a more iterative research process that better conforms to the complexity of behavior change [2–5].

The purpose of this paper is to define products and a preliminary process for efficiently and adaptively creating and curating a knowledge base for behavior change for real-world implementation. A central argument in our paper is for early-and-often sharing of resources developed—particularly operations/operational definitions—to facilitate more efficient knowledge accumulation for behavior change. To support early-and-often sharing, we specify a set of “products” from science that can be shared and then provide suggestions on a more “agile” scientific process for creating and sharing these products. Merriam-Webster defines agile as:” (1) marked by ready ability to move with quick easy grace <an agile dancer>; (2) having a quick resourceful and adaptable character <an agile mind>.” Some key synonyms include graceful, light, nimble, or spry. When we refer to agile, we are building on the second definition, particularly an adaptable and nimble scientific process.

To define these products and processes, we review evidence-based practice suggestions and draw parallels to software development. We then define three products—behavior change modules, computational behavioral models, and personalization algorithms—and a first draft research process called “agile science,” to produce these products. We then describe an ongoing case-study that has informed the agile science process. We conclude with open questions implied by the products and processes and suggestions for future work.

## EVIDENCE-BASED PRACTICE

Evidence-based practice involves the use of science to support pragmatic decision making related to the selection of behavior change interventions in a real-world context [1, 2]. Classically, evidence-based practices in the behavioral sciences grew out of a four-phase biomedical model (i.e., discovery, pilot, efficacy, and effectiveness), but there is increased questioning of this model because of the complexity of behavior change [1–7]. As implied by behavioral ecological models (e.g., [8]), individual and contextual differences (e.g., when, where, for whom, in what state of the person) can moderate when a behavioral intervention will produce its targeted effect [9]. Further, when developing interventions delivered via digital technologies, there is an added issue that the technologies are often mutating at a faster rate than the scientific process [3]. To complicate things further, behavior change interventions have multiple criteria for success for real-world use such as being effective, usable, desirable to the target group, and safe [5, 10].

There is a great deal of work focused on articulating research processes that are more appropriate for the complexity of behavior change. For example, recommendations related to multicomponent (also labeled “complex”) interventions increasingly emphasize an iterative and non-linear process [4, 5], and there is emerging discussion about different strategies for conducting reviews and organizing evidence [11, 12] to create evidence-based practice recommendations.

A particular advancement that we build on are “optimization methods” (see TBM special issue [13]). The organizing framework for these optimization methods is called the multiphase optimization strategy (MOST; [14]). MOST establishes a process for continuous optimization of behavior change interventions by iteratively evaluating the efficacy of intervention components. There are many optimization designs that fit into the MOST process. For example, the factorial/fractional factorial design provides a resource-efficient way of examining main effects of each intervention component within an intervention and the interaction effects among components [15]. The sequential multiple assignment randomized trial (SMART) is a methodology that can test the decision rules within an adaptive intervention, when, for instance, there are non-responders to an initial intervention [15]. A third class of experimental designs, which combines the logic of *n*-of-1 trials and factorial designs, is “micro-randomized” trial [16–18]. The micro-randomized trial is a sequential factorial design for modeling effectiveness of treatment components over time. It can be used to test proximal main effects of components over time, time-varying moderation [16], and with variations in study design based on strategies from system identification, the development of idiographic computational models [17, 18]. These optimization methods provide concrete strategies to test behavioral intervention components and, particularly via the micro-randomized study, support studying behavior change of individuals in context.

Another interesting suggestion is to take advantage of the “disruptive innovation” process when developing evidence-based practices [2]. The disruptive innovation process emphasizes the focus on providing simpler and less expensive alternatives to current practices that meet the essential need for the majority of users in order to make them more easily accessible, scalable, and replicable [2]. For behavior change interventions, suggestions from this include better synthesizing and sharing of modules of complex interventions that work across interventions, utilization of a wider range of delivery options (e.g., digital health technologies), the use of marketing and branding to promote and disseminate evidence-based practices, and the adoption of a continuous optimization model of evaluation. These recommendations were offered in contrast to dissemination of complex interventions that implicitly segment evidence-based practices rather than facilitate knowledge accumulation [19]. Indeed, Chorpita et al. [20] have argued that the fundamental challenge in behavior change science may be an overemphasis on knowledge proliferation (i.e., the development of new treatments) at the cost of knowledge management (e.g., developing new ways to design, organize, and share existing resources) and thus suggest focusing more on algorithms to curate knowledge.

From this work, we draw three insights: (1) development of evidence-based strategies for behavior change is iterative and involves “ongoing optimization” that carefully studies the “fit” between individuals, context, and interventions for producing a desired outcome [21]; (2) while complex interventions might be desired in practice, modules of complex interventions are plausibly more valuable for science; and (3) algorithms for supporting decisions about matching interventions and modules with specific individuals in context is a second valuable target product and could be useful for curation.

### Agile software development

These insights have interesting parallels to software development, particularly open-source software [22] and “agile,” which is a class of software development that emphasizes rapid iterative development in context [23]. For example, extreme programming (XP), which is one agile method [23], emphasizes well-specified modules that are shared early-and-often with relevant stakeholders when building complex multi-component systems. Within software development, the basic structure of modules is that they have specific inputs, a process that utilizes these inputs to transform them in some way and then this process creates specific outputs [24]. For example, a group may be interested in developing a smartphone app to promote physical activity and, as part of this, may be interested in using steps data from a wearable sensor such as the Fitbit. Based on this, one software module for this is a tool that allows data to be transferred between Fitbit and the researcher’s app that only shares information that the participant wants to share and protects the rest of the information such as personally identifiable information. A module that supports part of this data-sharing task while maintaining privacy is called “OAuth.” In OAuth, the inputs include information from the individuals (e.g., personal information such as name, email, etc.) and permission from the individual to allow information to be shared to another entity (e.g., when logging into a website, it may ask for a Facebook login, which has your personal identifying information). The OAuth process takes this information, generates a unique personal identification number for this individual, and then defines data that are sharable (e.g., steps data) versus not sharable between the systems (e.g., names, emails). The output of this process is a bridge for sharing only the information that a participant has agreed to share (i.e., a unique identifying number and way to link them to two separate software systems). It is essential to note that while modules are often built initially for a specific use case, an advantage of software module structure is that the module is built to be self-contained and thus repurposable for any use case with the requisite inputs (e.g., personal identification information, permission) and desires the requisite output (e.g., a secure bridge to share data across systems).

To create these modules, XP uses software “sprints” to create a “minimal viable product” (MVP) that is released to target users [23]. MVPs are the simplified modules that are designed to test assumptions about the module with relevant stakeholders [25]. Returning to the OAuth example, an MVP style test of this could simply be akin to the sorts of de-identification strategies used in research (e.g., separating personally identifiable information into a separate database from the rest of the data). This can then be used to test if privacy and sharing with other entities can be achieved prior to developing the software. This testing of assumptions via MVP modules is achieved via “sprints,” which involve a rapid cycle of identifying an assumption, building an MVP module to test the assumption, and then releasing the module to relevant stakeholders to test the assumption [23]. After each module achieves some meaningful criterion of success (e.g., privacy is maintained while still enabling sharing of data across systems to a satisfactory level for all stakeholders), the next module can then be targeted. While planning occurs, particularly specification of the likely required modules that will be needed for the eventual full system [24], the process emphasizes flexibility and agility to accommodate changing requirements depending on feedback received after each sprint.

Software development provides insights on three strategies to incorporate into health behavior change science: (1) define the smallest, meaningful, self-contained, repurposable, and (ideally) interoperable modules of an intervention (e.g., a module of a dietary intervention might be a fruit and vegetable tracker) that are structured in an input, process, output format; (2) build a concrete version of the module to make visible the implicit assumptions about how the desired outcome (e.g., higher vegetable intake) will be achieved by the module to enable discussion between stakeholders (note, preferably a functioning module, but a module could also be sketches, low-fidelity prototypes, and other strategies of communication used in human-centered design [26, 27]); and (3) use the shortest timescale possible to facilitate careful specification on the usefulness of the module for relevant stakeholders.

## THE CASE FOR GREATER EMPHASIS ON OPERATIONS

As with any scientific endeavor, we are mindful of the difference between abstract constructs and concrete operations [28]. In brief, constructs are abstract concepts or ideas that are often the generalized target of scientific research. For example, self-efficacy or goal-setting are both constructs. In contrast, operations are the concrete specifications (also called operational definitions) that are used within specific studies to define an abstract construct. To continue with the example, the specific strategy used to support goal setting in a specific study is the operation. The ultimate desideratum of science is robust generalized causal inferences that provide insights about the magnitude and direction of interrelationships between constructs [28]. For example, the insight that positive reinforcement is a useful intervention strategy for behavior change across a wide range of behaviors, types of target populations (including different species), and settings is the sort of ideal insight to strive for [29]; a point we are not contesting. Meaningful products from any scientific endeavor include well-specified operations, constructs, and/or causal inferences, particularly causal inferences with high external validity [28]. Greater value should be placed on concrete operations, even if they have not been empirically validated because: (1) operations are highly valuable for better specifying constructs; (2) early sharing of these operations could very likely enable a more efficient research enterprise via reduced “reinventing the wheel” and facilitation of specialization; and (3) sharing of operations enables different research groups to use the same operations in different use cases, which enables a more robust testing of external validity.

In terms of construct specification, concrete examples, particularly multiple examples, provide richer information about a construct than a textual definition alone. For example, a practitioner may be interested in using the construct “cue to action” to inspire behavior change. A cue to action could be defined as a signal that elicits a behavioral response. This abstract definition requires a great deal of interpretation before it can be used, particularly by non-behavioral scientists (e.g., clinicians, software developers). Another way to describe a cue to action is to provide concrete examples (e.g., a bar can be a cue to drink, the smell of smoke can cue the urge to smoke, a calendar notification can cue the action of getting up to go to a meeting, a feeling of stress can cue the urge to go for a walk). Often multiple concrete examples are complementary to abstract definitions as they provide subtle details missed in the construct definition. Consider, for example, current debates about if Pluto is a planet; the debate revolves around the match between the text definition of the construct of a planet and the specific instance of Pluto. A logical product from agile science should be multiple concrete operations of constructs that are shared early and often to facilitate more effective debate about construct definitions.

With regard to a more efficient research process, a logical corollary for this is the technology industry’s use of application programming interfaces (APIs) [30]. APIs are the basic instructions software developers create to allow other developers to share tools and resources. For example, Yelp displays maps of restaurant locations. Yelp did not create a mapping service but instead took advantage of Google Maps’ API, which allowed Yelp to “embed” Google Maps into their service. Google defines the inputs it will need (e.g., a street address) and the output it will provide (i.e., maps, directions) in a prespecified format (e.g., the window size for the map). It then processes the inputs to produce the output. When the API code is incorporated into the Yelp code, the map appears within Yelp’s products. This example is illustrative of our two points related to efficiency. First, while Yelp requires a strategy for displaying locations of restaurants to their customers, they did not need to “reinvent” a mapping service; instead, they just used Google’s already available service. Beyond this, arguably, Google Map’s API was an essential component that Yelp required prior to even starting. Otherwise, the developers working at Yelp would have invested energy into the mapping service, which would have limited their ability to specialize on their unique service of restaurant recommendations.

It is plausible that similar value of reducing reinventing tools and specialization could occur in the behavioral sciences if we shared our operations early and often, even prior to formal evaluation. It has been argued elsewhere that evidence-based practices often become their own separate silos of evidence, rendering it very difficult to mix and match modules across evidence-based practices [2]. A plausible reason for this siloing of evidence-based protocols is simply that we are sharing our operations at the wrong time. We argue that sharing operations PRIOR to effect size estimation (e.g., prior to running an efficacy trial) could greatly mitigate this problem. Put differently, sharing early and often enables individuals to build on one another’s work at the beginning and thus makes it easier for the process to facilitate interoperable and repurposable modules across complex interventions. This reduces independent groups reinventing similar operations and also, if the information is well-curated and modules are interoperable, could facilitate increased specialization.

On the advantages to external validity, as articulated by Shadish, Cook, and Campbell, external validity involves examination on how likely a given causal inference is true across variations in the target population (also labeled “unit”), treatment, outcome, and setting being studied [28]. As this example illustrates, good external validity requires variations in who uses an intervention, how the intervention might be operationalized, the outcomes the intervention is being targeted to influence, and the settings in which the intervention is used. This type of data could more easily be gathered for the same or similar operations if operations related to behavior change interventions were released early and often and then incorporated into different research projects for different populations, settings, or outcome measures. At present, many of our operations, particularly our interventions, are often treated as the “secret sauce” of our research and only shared after years of evaluation. As a single research group often does the evaluation, this results in limited variability related to populations, treatments, outcomes, and settings, thus providing limited data for external validity claims. We recognize the challenge we are making to cultural norms but hope that these plausible advantages of early-and-often sharing might help spur discussions on how to shift the culture (see “Future directions” section).

## AGILE SCIENCE PRODUCT TYPES FOR BEHAVIORAL INTERVENTIONS

With the value of sharing operations specified, there are three behavior change intervention product types that we view as particularly important for targeting as they are the conceptual building blocks for personalized complex interventions: behavior change intervention modules, computational models, and personalization algorithms. Prior to specifying these though, it is important to note that other products such as good operations and constructs related to target populations and settings are also valuable. We explicitly focus on product types that are needed to directly facilitate behavior change but other work should examine how best to better specify and share other relevant operations and constructs.

We define behavior change modules as strategies designed to produce a specific and scoped behavioral outcome. The sort of scope we are envisioning is most akin to behavior change techniques in that they are meant to be irreducible components of a behavioral intervention [31]. The modules are designed to produce a specific outcome (e.g., have a person set a goal) which, when combined with other modules, can produce more distal outcomes (such as supporting weight loss). There is advantage to structuring modules using the software module structure of inputs, processes, and outputs. As the API example illustrates, Google requires specific inputs (e.g., address), conducts a specific process (e.g., linking this address to their map database), and produces a specific output (e.g., a map). Within the behavioral sciences, behavior change modules could conform to this generic structure to support better “system architectures” of the intervention [24]. For example, a goal-setting intervention often requires inputs such as behavioral history or preferences, conducts a basic process (e.g., translates past behavior and preferences into a target goal), and produces an output (e.g., the behavioral goal for a person). Careful delineation of inputs, processes, and outputs makes it easier to draw connections between behavior change modules and thus the architecture on how modules interact (including modules for measuring attributes of the person/population, setting, or outcome measure but, again, those modules are not the focus of this paper). For example, a goal-setting intervention requires an input of past behavior. This past behavior could come as the output of a self-monitoring intervention. On the flip side, the goal output could be incorporated as an input into other modules such as a social leader board to enable social comparison.

Four adjectives—smallest, meaningful, self-contained, and repurposable—provide criteria for a “good” module. The concepts of meaningful, self-contained, and repurposable have self-evident value but the value of smallest might be less obvious. In brief, striving for small modules enables repurposability. Often a tool is developed for a very specific use case. This use case often includes features that are thought as central to that module’s purpose. As more features are added, the module becomes increasingly idiosyncratic to the specific use case. For example, a blog post from the Center for Behavioral Intervention Technologies at Northwestern University describe a problem like this for “Purple Robot,” which is a sensing system used for behavioral research [32]. The team recognized that Purple Robot needed to “go on a diet” as there were too many features limiting repurposability. According to the blog, the team distinguished core features versus “add-ons” to increase repurposability. This example illustrates the value of the smallest meaningful self-contained module to increase repurposability.

The second product type are computational behavioral models, which we define as mathematically defined versions of behavioral theories that require greater specification than current behavioral theories. In particular, behavioral theories classically define model structure (i.e., how variables are related to one another) and predictions about directionality and magnitudes of effects of interventions on an outcome. A computational model includes these specifications but also includes (1) relationship dynamics, including issues such as timescale of an effect [33, 34], response patterns (e.g., a linear or non-linear response [35]), latency, and decay [33]; and (2) the boundary or “threshold conditions” that define when, where, for whom, and in what state of the individual an intervention will produce the desired effect [9].

The complexity of behavior change, as implied by complex interventions [5], often requires an understanding of how modules interact with the individual, context, and with each other over time, particularly to achieve a clinically meaningful outcome (e.g., moving a person from sedentary to 150 minutes of moderate activity). Behavioral theories have classically been how behavioral sciences describe how interventions, individuals, and context interact but these are fraught with well-documented problems such as lack of falsification, lack of information about subtle dynamics, and they are often so abstract that they are difficult to apply in practice [36, 37]. The computational model is the agile science equivalent of a behavioral theory as it specifies how persons, interventions, outcome targets, and setting interact to produce a target outcome. We envision that computational models will take the form of dynamic system models [17, 18, 33, 38], time-varying effect models [16], or other such dynamic models.

**Fig 1:**
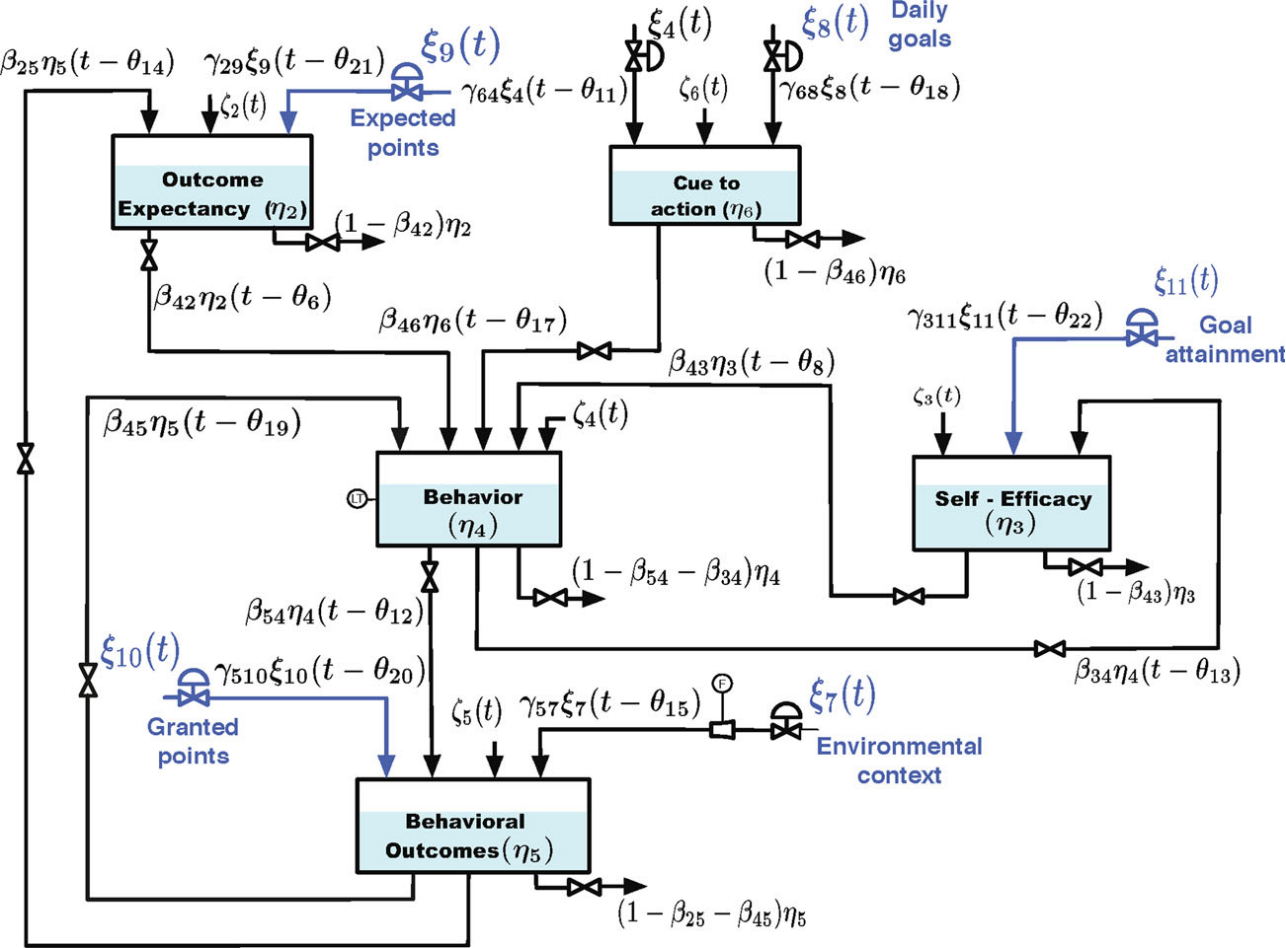
Computational model structure of a just in time adaptive intervention. Adapted from Martin, Deshpande, Hekler, and Rivera [38]

We are extending the concepts of operations and constructs to theoretical frameworks in the form of computational models. This is to acknowledge the common problem that there is often a difference between the complete abstract theory defining all plausible variables and interrelationships (a theory-construct) versus the concrete implementation of the theory in a specific study that does not include all of the variables has specific operations for each variable, and, by extension, often only examines a subset of the variables and interrelationships (theory-operation). For example, Fig. 1 is a computational model operation, specifically a mathematically specified dynamical model that is being used for a specific intervention implementation [17, 38]. This computational model operation was derived from the computational model construct of a full dynamical model of social cognitive theory described elsewhere [39].

**Fig 2:**
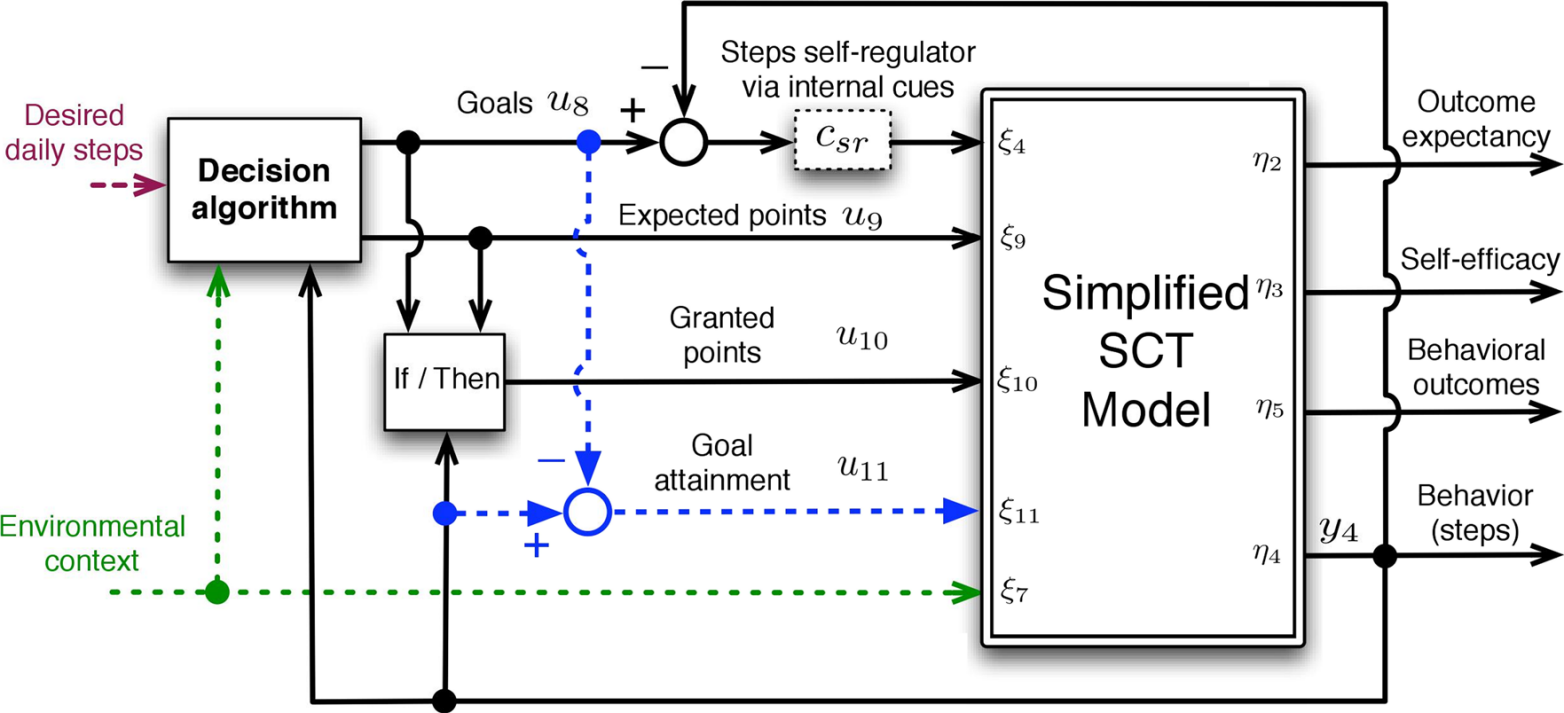
Diagram of the model predictive controller that utilizes the computational model in Fig. 2. Adapted from Martin, Hekler, and Rivera [43]

Computational models can be generated using simulations prior to collecting data [39]. For example, Riley et al. utilized simulation to generate the computational model theory-construct of social cognitive theory [39]. Specifically, the team generated a computational model of social cognitive theory and simulated known behavioral phenomena such as habituation. These simulations from the computational model produced predictions that were then examined against expectations. This computational model simulation was then further simplified to make it possible to operationally define in a study (Fig. 2). Specifically, the simplified computational model operation mathematically defines how specific intervention modules (e.g., an adaptive goal-setting module), the person (e.g., self-efficacy, stress, busyness), and environment (e.g., location, weather, day of the week) interact to define an ambitious but doable step goal. This computational model was generated via simulation [39] and was designed to be empirically validated via system identification [17].

Our third product is a personalization algorithm, which we define as decision rules, including computational tools that translate information from computational models into intervention selection decisions. Personalization algorithms provide an answer to the question: “Which behavior change intervention(s) should be used for this person (or other target unit such as school, community, city, etc.) at this time and context to achieve the desired outcome?” As such, the personalization algorithm is a pragmatic target for developing evidence-based recommendations of complex interventions as the personalization algorithms establish the evidence base for selecting the right intervention option for a particular person in context. Model predictive control [40–43], recommender systems [44], agent-based modeling [45], or Bayesian network analysis [46] are all plausible personalization algorithm strategies.

Personalization algorithms serve two functions. First, they can dynamically determine which intervention option available in an intervention needs to be used for a specific user at a specific time. For example, in the system dynamics framework, a model predictive controller translates the knowledge contained in a computational model into specific adaptation decisions [43]. The computational model may provide predictions on the dynamic interplay between variables (e.g., how weather, stress, busyness may each contribute to what is ambitious but doable for a person), but this information must ultimately be translated into decisions that a system could enact such as selecting a step goal on any given day for each person. The model predictive controller utilizes the available knowledge codified in the computational model and other information such as noise in the model (see Fig. 2) to make such decisions.

The second role of personalization algorithms is to aid the selection of intervention modules that might be effective for individual people in their specific context. Such intervention personalization might be accomplished through recommender techniques [44], such as the ones used by companies such as Netflix and Amazon (e.g., since you liked the Avengers, you might like Iron Man 3). A recommender system could utilize demographics (e.g., age, gender, personality characteristics) to make recommendations on intervention modules appropriate for a person (e.g., women aged 40–60 just starting exercise tend to do best in groups; here are some groups that you can join in your area). The ability of recommender systems and similar algorithms to suggest appropriate intervention modules depends on the accumulation of data that clearly includes variations in persons, intervention modules, outcomes, and settings to enable effectve matching of particularly interventions for particular persons to achieve a desired outcome. Early-and-often sharing is thus a prerequisite for the accumulation of data needed to power such intervention personalization.

In summary, we argue for recognizing a wider range of scientific products, including the three key product types we described above: behavior change modules; computational models; and personalization algorithms and also argued for recognizing the value of both operations and constructs independent of causal inferences. Together, these three agile science products each uniquely contribute to supporting evidence-based decision making about which intervention to use for whom and at what time.

### Agile science process

**Fig 3:**
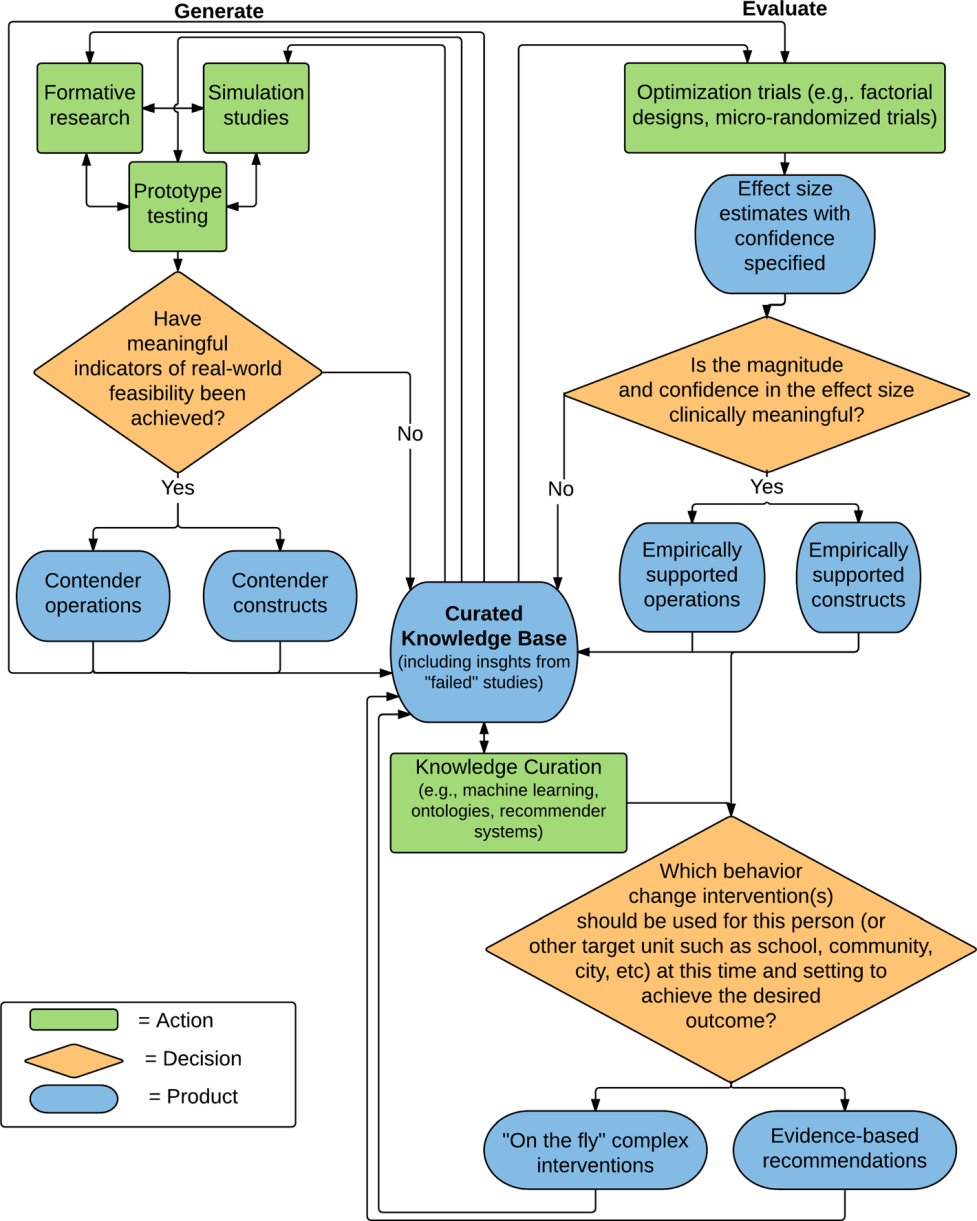
Agile Science Process v0.1

We propose a first draft process (v0.1) to produce and share these products early and often (see Fig. 3). It is essential to note that the agile science process starts and ends with the curated knowledge base, which we envision includes a much wider range of insights and resources than is currently common in the scientific literature. Extending the argument of Chorpita et al. [20], it is essential to acknowledge the essential role for effect curation of knowledge, which is a separate complementary process to creation. For the purposes of this paper, we focus on products and a process for knowledge creation as we view careful delineation on what to generate as a logical prerequisite prior to curation but future work should better articulate curation strategies most useful for real-world behavior change interventions. There are two complementary and iterative phases, the “generate” phase and the “evaluate” phase. Examining the emphasis of human-computer interaction (HCI) versus behavioral science is instructive for distinguishing the phases. Within HCI, there is an emphasis on novel technology tools and artifacts, not necessarily reliable effect size estimates/causal inferences [47]. Within the behavioral sciences, reliable effect size estimates for constructs are more important to support evidence-based decisions [1]. Within agile science, we see these two as complementary with, roughly, HCI processes conforming to the generate phase and behavioral science processes roughly conforming to the evaluate phase, though obviously there is overlap.

The purpose of the generate phase is twofold: (1) to produce multiple contender operations to specify contender constructs of modules, computational models, and personalization algorithms; and (2) to conduct feasibility testing for real world use using the criteria from Bowen et al., including acceptability, demand, implementation, practicality, adaptability, integration, and limited efficacy [10]. By contender, we explicitly acknowledge that the products produced via the generate phase are not evidence-based. The contender label is used to minimize inappropriate claims about effectiveness for the sorts of small trials recommended in the generate phase [48] but also to acknowledge their value. The generate phase includes formative work, simulation studies, and prototype testing.

The goal of formative work is specification of the problem, goal, population, and setting and ideation on plausible solutions to achieve targeted goals. It includes methods such as human-centered design [27] and community-based participatory research [7] and also explicitly includes careful review of previous accumulated knowledge. These methods are acknowledged as critical for a wide range of evidence-based practice recommendations related to the research enterprise [5].

The goal of simulation studies is to specify computational models and also plausible personalization algorithms with limited or no data. As discussed earlier, Riley et al. utilized simulation to generate a computational model of social cognitive theory [39]. The simulation study process is, by definition, highly iterative and involves specifying the attributes of a computational model (i.e., model structure, predicted magnitude, direction, and dynamics of interrelationships, and the interaction between individual and contextual differences on when the intervention will produce an effect). As each of these features are specified, the developer can then run a simulation to examine if the predicted output from the simulation is in line with their expectations. For example, in the Fig. 1 dynamical model, the team has conducted a series of simulations to explore different ways of defining an “ambitious but doable” goal based on interactions between the variables in the model. This sort of process is highly valuable as it allows for complex and dynamic hypotheses to be mathematically specified and thus testable.

The goal of prototype testing is twofold: (1) to facilitate further specification of operations via the process of building an operation and (2) to do feasibility testing by sharing prototypes with relevant stakeholders to receive feedback. For early testing of feasibility in the generate phase, we see particular value of “lean start-up” methods [25]. In brief, lean start-up methods provide strategies for identifying assumptions about an eventual product or service and then devising the most “lean,” which means resource-efficient, strategy possible for testing the assumption. For example, the founders of Dropbox made the assumption that there would be demand for a service that makes it easy to sync and share files. To test this assumption, they could have built the technology but that would have required considerable resources. Instead, they tested their assumption with a video (http://techcrunch.com/2011/10/19/dropbox-minimal-viable-product/). The video was a test on the feasibility of the idea (e.g., acceptability, demand) that was resource-efficient as no technology was developed. A central future area of research is to systematically study how best to translate this type of lean process into a scientific process that is useful for testing feasibility issues for behavior change interventions (see “Future directions” section).

It is essential to acknowledge the highly iterative and dynamic nature of the generate phase. It is likely that a researcher engages in formative work, simulation studies, and even prototyping testing iteratively and/or simultaneously. For example, a prototype test of a problem-solving module might reveal the need for better problem or target user specification, thus the need for more formative work. Alternatively, the team may be interested in a complex outcome such as weight and thus do simulation studies to specify dynamic predictions, while actively prototyping and doing formative work. As something is learned with one action, that information is fed into the others until contender operations and constructs emerge that show promise for being useful in the real-world.

Contender operations and constructs move to the evaluate phase. The purpose of the evaluate phase is to develop robust generalized causal inferences via effect size estimates. We build on the logic of optimization methods from MOST [14]. The core output of the evaluate phase are the classic targets of behavioral science including effect size estimates and confidence/credibility intervals of the interrelationship between constructs. Particularly when methods such as micro-randomized trials are used, they provide a resource-efficient way to evaluate the agile science product types as data will be available about the interrelationship between modules, individuals, and context over time [16]. If clinically meaningful effect sizes are found, then a target product can be relabeled from a contender to an empirically supported product. These empirically supported products, particularly when data are pooled to support recommender system processes discussed earlier, can then provide an evidence-based answer to the question: “Which behavior change intervention should be used for this person (or other target unit such as school, community, city, etc.) at this time and context to achieve the desired outcome?”

After both the generate and evaluate phases, all products created are shared into the curated knowledge base including “failed” studies such as information about how a contender operation did not appear to have real-world feasibility from the generate phase or operations that did not appear to show clinically meaningful effects from the evaluate phase. As Fig. 3 illustrates, we explicitly argue for early-and-often sharing of all products and insights from failed studies generated within the scientific process into the curated knowledge base. As described above, contender operations are the materials needed for exploring issues of feasibility and also for carefully specifying constructs. The classic four-phase model emphasizes effect size estimates prior to testing feasibility in an effectiveness trial [7]. As the Dropbox example illustrates, formative research methods, simulation studies, and prototype testing can examine feasibility prior to development of final operations, let alone constructs, or causal inferences. This is important as the agile science process explicitly places questions of feasibility and construct specification as equally important to effect size estimation as represented in the generate phase. Many of the tools already exist for early-and-often sharing of contender operations for digital health interventions such as open-source code repositories (e.g., GitHub), open science forums (e.g., Open Science Framework), and emerging open infrastructures (e.g., [49]).

As our goal is evidence-based practice, the process is focused on answering the question: “Which behavior change intervention(s) should be used for this person (or other target unit such as school, community, city, etc.) at this time and context to achieve the desired outcome?” Modules, computational models, and personalization algorithms provide the evidence-based “building blocks” for generating practice recommendations for combining modules into complex interventions for unique use cases. An interesting opportunity that could emerge is a new type of complex intervention, one that might be called an evidence-based “on-the-fly” complex intervention. Specifically, on-the-fly complex interventions involve the evidence-based selection of appropriate modules for an individual that are selected specifically for that person. This type of on-the-fly complex intervention is a logical target that is in line with the prevention and treatment strategies being called for within the Precision Medicine Initiative [50].

### Ongoing case study of agile science

Multiple complementary research projects are underway that each focus on developing a different module for a complex intervention for physical activity. These research streams were largely the inspiration for v0.1 of the agile science process and thus are informative to help make the potential of agile science more concrete. The work already described in defining an ambitious but doable step goal is one such project. From an agile science perspective, our goal is to develop a module, computational model, and personalization algorithm that define ambitious but doable step goals for a person in context and over time to support physical activity. Combined, this could function as an effective goal-setting module that could be used in a complex intervention. A sister project headed by coauthor Klasnja, is focused on developing modules that support physical activity maintenance [16]. In brief, the focus of this work is on the development of two intervention strategies: (1) providing context-relevant cues to engage in physical activity (e.g., it is the morning, sunny, and you have time open in your schedule; up for a walk?); and (2) daily planning (e.g., when, where, and how an individual will be active).

A third complementary project is focused on the development of a tool that can support the rapid creation and early-and-often sharing of behavior change modules. In partnership with Bob Evans at Google and his open-source Paco system (www.pacoapp.com), at the time of writing, we are building a system that allows researchers to rapidly build behavior change modules. The focus is on developing a process for doing rapid prototyping and early-and-often sharing of the behavior change modules.

Within the ambitious but doable and context cue/implementation intention projects, we previously engaged in formative work, simulation studies, and prototype testing and thus are currently utilizing the operations created previously in the evaluate phase. When combined, our hope is that the modules generated across these projects will enable on-the-fly complex interventions.

## DISCUSSION

Great advantage for evidence-based practice can occur if we, as a field, embrace the value of a much wider range of contributions to scientific practice beyond just effect size estimates. In particular, behavior change modules, computational models, and personalization algorithms are the building blocks for creating complex interventions and thus are logical scientific targets. Further, sharing contender operations and constructs and also failed tests of these contenders from early work has great value for supporting better construct specification, improved efficiency in the scientific process, supporting earlier tests of feasibility, and could plausibly support better collection of data related to external validity and, by extension, support recommender systems for personalization algorithms and knowledge curation. The first draft (v0.1) of the agile science process includes both the generate phase and evaluate phase that both end in sharing these products and insights in the curated knowledge base. It is a starting structure for how to create, share, and evaluate these products. Our work raises many questions.

It might seem counterintuitive to focus on modules when tackling something as complex as behavior change. The incredible array of software developed modularly (e.g., Apache, arguably the foundation of the Internet) provides evidence for this approach, but there are also epistemological reasons. In brief, targeting modules, when complemented with computational models and personalization algorithms, is a reduction strategy that does not ignore the complex nature of behavior. In contrast, the four-phase development model reduces the complexity of the problem by assuming that a topic can be studied out of context (e.g., efficacy trials) to increase internal validity prior to external validity (i.e., effectiveness trial). As such, the four-phase model ignores variations of units, treatments, outcomes, and settings until the end of the process but it is highly feasible that when, where, for whom, and in what state of the individual can all moderate when an intervention will produce an effect [9]. Focusing on modules, building in context, and updating fit embraces these variations and can be codified in computational models and personalization algorithms.

Another important question is the role of randomized controlled trials (RCTs) of complex interventions. The RCT does have an important role but it must be used when the research question dictates its use. We see at least three use cases: (1) quality assurance of a complex intervention that will be released “as is” to a large population; (2) as an “A/B” style test that is often used in the tech industry; and (3) as used in the “trials of principles” [51]. RCTs of complex interventions are the most logical method for establishing that a specific complex intervention will work for a specific target population. An investment in an RCT of a complex intervention is warranted whenever there is a large enough population that will use a standardized protocol over an extended period of time without updating the protocol. For example, if a health maintenance organization plans to have two million individuals use a complex intervention, conducting an RCT with a representative sample is appropriate.

The second use is A/B testing [36]. In the digital technology industry, there is a common practice of comparing different versions (often two, hence A/B) of a given system. The two versions represent different hypotheses on how the system might function and are often only subtly different. For example, Bond et al. conducted a 61 million person A/B-style experiment on Facebook to explore how message framing might influence voting behavior [52]. There were three conditions: the information condition, which provided information about voting; the social condition, which included everything in the information condition and images of friends that voted; and a control group that did not receive any information. As this example illustrates, the A/B test uses the logic of an RCT but is focused on small differences. As such, it is more akin to the optimization methods discussed earlier. The third use case for RCTs is the trials of principles, which emphasizes that in behavioral intervention technologies, it is common for a software to change over time but the general principle (e.g., using goal setting as a technique) does not. Within a “trials of principles” RCT [51], the focus is on ensuring the test is on those principles (i.e., the module constructs to use our labels in this paper), while allowing non-tested parts of the system not deemed principles to be updated over time. In a trial of principles, heterogeneity between conditions is minimized with careful comparator selection and the method does not ignore technology changes. As such, it fits well with agile science within the evaluate phase.

While the agile science process likely makes the most sense within digital health interventions, can it be applied to other intervention mediums? We believe it is quite plausible that the same process can work for other intervention modalities (e.g., face-to-face, group) and other types of complex problems (e.g., precision medications, public policy initiatives). As with any scientific process, the essential requirement is replication with sound definitions, robust versioning control, and a process of rapidly sharing and curating that information. This is easier with software but can be difficult with other modalities. That said, striving toward better-specified operations and constructs, replication, and more efficient curation is possible with non-technology interventions. Behavioral science has many examples of carefully replicated experimental manipulations (e.g., careful specification of cognitive dissonance [53]). The major limitations are intervention specification to support replication, which is ideal for all sciences and effective curation as already pointed out by others [20].

### Future directions

More research is needed on early-stage work, which we label the generate phase. The generate phase produces the contender operations and constructs and provides insights about real-world feasibility that then can be tested in the evaluate phase. Previous work suggests early studies often test feasibility of methods (e.g., recruitment, randomization, retention, and assessment), not hypotheses [48]. Other recommendations suggest a clear distinction between testing feasibility of the operations versus research methods [54]. Future work should be conducted to assess how the early-phase process could be adjusted to better enable the generation of multiple contender operations, initial filtering of these operations related to issues of feasibility, and careful, but plausibly separate, testing of the research methods themselves for a later trial, when that is appropriate.

Further work is needed on devising resource-efficient experimental designs and statistical analyses for the generate phase, particularly to support decision making on if rigorous evaluation is warranted. For example, sequential *n*-of-1 trials could serve as a more efficient feasibility testing method by allowing for intervention adjustments after each series of trials [55]. From a human-participants perspective, micro-randomization trials are very efficient and thus could also be used after more classic *n*-of-1 trials have provided enough confidence to warrant investment in the design of one [16]. In terms of statistics, a Bayesian approach might be more appropriate than Fisherian statistics [56] as the Fisher approach is not appropriate with limited data [48]. As demonstrated elsewhere, careful use of priors within studies of novel systems can be used to help mitigate problems with magnitude effect errors and thus could be a more appropriate statistical method for the generate phase [47].

With regard to idea generation and vetting, there are important cognitive biases that likely come into play that need to be better acknowledged in the generate phase. For example, Dow and colleagues showed the possibility of developing better results through iteration [57] and a subsequent study examined the differences between parallel and iterative development versus serial and iterative development [58]. Results found that individuals randomized to work on multiple ideas at once (i.e., parallel group) had greater openness to incorporating feedback and also produced better end products compared to the single iterative concept group. Individuals in the single concept group were less open to feedback and instead often “stayed the course.” This stay the course observation is similar to the sunk cost bias [59], which is a well-known cognitive bias that suggests an individual will pursue a less desirable option even if a more desirable option becomes available because of the resources already invested. The substantial fiscal and intellectual cost of the current research enterprise may make scientists prone to the sunk cost bias thus stymying changing course when it might be warranted. More rapid and parallel development could support both better defining of a construct and minimize the impact of the sunk cost bias, thus further reinforcing the value of early-and-often sharing and developing multiple operations.

Finally, it is essential to continue exploring how the research enterprise might enable or stymy the creation and early-and-often sharing of products from science, particularly operations [3, 60]. As suggested by Ioannidis, it is quite plausible that the current reward system for academics, particularly the currency (i.e., publications and grants), may be resulting in the unintended consequence of incentivizing the creation of only minimally useful products for real-world use. Further exploration on the impact of the design of the research enterprise is warranted that not only explores how to minimize bias (e.g., the sunk cost bias) but also articulates how the incentive structures of science can be better aligned to useful products for real-world use. For example, early-and-often sharing is not well incentivized in the current research enterprise, particularly the products from the generate phase. That said, it could very likely improve the scientific community’s ability to create efficient accumulation of knowledge about behavior change. Future work should study incentives for academics that reinforce early-and-often sharing. In addition, we further reinforce Chorpita et al.’s call for more focus on effective knowledge curation [20].

## SUMMARY

In this paper, we established three products: modules, computational models, and personalization algorithms, as logical targets for supporting more effective and efficient knowledge accumulation for evidence-based practice. We then presented a rough outline of an agile science process that emphasizes more scientific products to share early and often, including operations and also failed tests. As our ongoing suite of complementary projects illustrate, we are actively working toward testing the assumptions outlined. We see our suggestions as complementary to the many other suggestions for improving the efficiency of knowledge accumulation for evidence-based practice.
